# Concizumab, a Non-Replacement Therapy for Persons with Hemophilia with Inhibitors

**DOI:** 10.3390/jcm14092961

**Published:** 2025-04-25

**Authors:** Giancarlo Castaman, Victor Jimenez-Yuste, Johnny Mahlangu

**Affiliations:** 1 Center for Bleeding Disorders and Coagulation, Department of Heart, Lungs, and Vessels, Careggi University Hospital, Largo Brambilla, 3, 50134 Florence, Italy; 2 Haematology Department, Hospital Universitario La Paz-IdiPaz, Autónoma University, Paseo de la Castellana, 261, 28046 Madrid, Spain; 3 Department of Molecular Medicine and Haematology, Faculty of Health Sciences, University of Witwatersrand, 7 York Road, Parktown, Johannesburg 2193, South Africa

**Keywords:** concizumab, hemophilia A, hemophilia B, inhibitors, non-replacement therapy, rebalancing agents, tissue factor pathway inhibitor, unmet needs

## Abstract

Despite enormous progress in the development of therapeutic agents for persons with hemophilia A and B (HA, HB), several unmet needs persist. These are disease- and treatment-related. Prophylaxis with clotting factor replacement is the gold standard, but not feasible in HA and HB with inhibitors. Whereas persons with HA with inhibitors can receive prophylaxis with a factor-mimicking agent, emicizumab, there is no recommendation for the agents to use as prophylaxis in persons with HB with inhibitors as there are no available molecules. Concizumab is a novel, subcutaneous prophylaxis option in persons with HA or HB with inhibitors that can potentially improve long-term outcomes. Here, we review the available data on concizumab and discuss its possible positioning in the armamentarium to treat hemophilia with inhibitors.

## 1. Introduction

Hemophilia A and B (HA, HB) are rare, congenital recessive chromosome X-linked bleeding disorders in which clotting factor VIII (FVIII) and IX (FIX), respectively, are lacking or deficient [1]. Both factors are part of the intrinsic coagulation pathway involved in the amplification phase of clotting [2]. An FVIII and FIX deficiency compromises factor X (FX) activation, leading to suboptimal thrombin generation and the inadequate strength of the early clot [3]. Thus, especially in the absence of treatment, persons with severe forms of HA and HB suffer from spontaneous bleeds into major joints and intramuscular bleeds and possible intracranial hemorrhages with consequent comorbidities, whereas persons with a moderate or mild disease may experience post-traumatic or surgery-related bleeds [1]. Joint bleeds are a serious complication of hemophilia leading to disability [4].

The replacement of the deficient clotting factor continues to be the gold standard of therapy for hemophilia [1,5,6]. Replacement therapy can be delivered through prophylaxis or ‘on-demand’. Prophylaxis aims at raising background levels of circulating coagulation factors to prevent spontaneous bleeds and results in better outcomes than treatment on-demand [6]. Treatment-related unmet needs remain. Recently, there has been growing interest in developing treatment strategies that do not involve deficient clotting factors. Accordingly, several non-replacement approaches are being explored. These include factor mimetics or rebalancing agents, such as antithrombin inhibitors, activated protein C inhibitors, and tissue factor pathway inhibitors (TFPIs).

Concizumab is a monoclonal antibody that inhibits TFPIs and was the first drug of its class to be approved by the regulatory agency in Canada [7]. Late in 2023, concizumab made it onto the list of “antibodies to watch in 2024”, an annual summary of therapeutic monoclonal antibodies in late stages of clinical development, a regulatory review, or those for which a first approval was recently granted [8].

This narrative review aims to summarize the available data on concizumab and map its place in the context of the unmet needs of persons with HA and HB with inhibitors and the existing treatments for the two bleeding disorders.

## 2. Unmet Needs

The primary unmet need in hemophilia, regardless of whether HA or HB, is the possibility of living a normal life without the constraints of the disease and its treatments [9]. None of the currently available therapies can ensure a fully normal life for persons with hemophilia. Although prophylaxis with factor replacement therapy is the standard of care, it has its limitations. Replacement therapies have been linked to an important treatment burden due to the frequency of intravenous infusions needed (partially overcome by the development of extended half-life products), the need for intravenous access, treatment-related pain, lengthy infusions, fridge storage, and the logistics of organizing everyday life and work around the injections, to name just a few issues [5,10,11]. When treated with replacement factor concentrates, some patients develop neutralizing antibodies (inhibitors) against exogenous clotting factors. Such inhibitor development occurs in up to 30% of persons with HA and up to 10% of persons with HB [1,6,12,13,14,15,16], and results in the inactivation of infused factors, the difficult management of bleeding episodes, and a greater disease burden [6,17,18,19,20].

Persons with HA and HB with inhibitors are the two populations for which few therapeutic options are available. For persons with HA with inhibitors, there is an unmet need for treatment options that prevent bleeds to be used concomitantly with on-demand treatments without additional safety concerns. An activated prothrombin complex concentrate (aPCC) and emicizumab are the only approved drugs for prophylaxis in the setting of HA with inhibitors [6,21,22]. However, the greatest and most urgent need for novel treatments is for persons with HB with inhibitors [23]. Despite extensive evidence on the general benefits of prophylaxis, the World Federation of Hemophilia has no recommendations for persons with HB with inhibitors [6]. Immune tolerance induction is possible but difficult and costly, and it fails in up to one-third of individuals [16,24,25]. In fact, persons with HB with inhibitors may have anaphylactic reactions to products containing FIX [6]. The development of inhibitors is also an exclusion criterion for gene therapy eligibility [26]. Gene therapy has the potential to transform treatment, but it is not available for all persons with hemophilia. Besides persons with HA and HB with inhibitors, individuals with pre-existing immunity against viral vectors used for gene delivery are also currently ineligible for gene therapy [27]. Moreover, there are uncertainties concerning the duration of transgene expression, with the factor expression declining over time [28], and the long-term safety of such approaches, particularly regarding liver toxicity and genotoxicity [29].

The limitations of traditional factor replacement therapy led to research into non-replacement treatments, taking advantage of varied mechanisms of action. Emicizumab was the first non-replacement treatment approved by regulatory agencies worldwide for routine prophylaxis in persons with HA with inhibitors and persons with moderate-to-severe HA without inhibitors [30,31]. Emicizumab is a bispecific monoclonal antibody that bridges FIXa and FXa to restore the hemostatic function. Its development filled the gap in therapy for persons with HA with inhibitors, but emicizumab cannot be used in persons with HB with inhibitors. Although long-term study outcomes confirmed low annualized bleeding rates (ABRs) and no new safety signals, real-world data reported a variable incidence of bleeding episodes, some severe, in persons with HA on emicizumab prevention [32,33,34,35,36]. Although emicizumab has minimal immunogenicity, several cases of neutralizing anti-emicizumab antibodies in persons with HA without FVIII inhibitors have been described [37]. Taken together, these data show that there are still unmet needs in persons with hemophilia with inhibitors. Hemostatic rebalancing agents may be able to meet these needs.

## 3. Rationale for Inhibiting TFPIs

Hemostasis is a complex process that ensures blood flow and prevents losses after injury [38,39]. For the correct functioning of the hemostatic system, there must be a balance between natural procoagulants and anticoagulants; any imbalance can lead to either pathologic bleeding or thrombosis [40]. Natural procoagulants are the clotting factors, whereas anticoagulants include antithrombin, TFPI, heparin cofactor II, protease nexin 1, Z-dependent protease inhibitor, and activated protein C [3,40]. The synergistic modulation of the tendency to bleed by anticoagulant and fibrinolytic factors was described in persons with hemophilia, in whom a deficiency of those factors was associated with a milder bleeding phenotype [41]. The inhibition of natural anticoagulants can restore hemostasis in patients with bleeding disorders [42].

Among natural anticoagulants, the TFPI, a multivalent Kunitz-type proteinase with an inhibitor, prevents the unrestricted amplification of the clotting cascade. It inhibits not only FXa, but also activated factor VII (FVIIa), thereby avoiding FXa generation by the extrinsic clotting pathway [43]. TFPI inhibition allows for sustained thrombin generation, despite an FVIII or FIX deficiency in persons with HA/HB [3]. Thrombin generation is crucial for blood coagulation: it converts fibrinogen into fibrin to form a clot. Thrombin is generated by FXa, which, in turn, is activated either by the tissue factor/FVIIa complex or by the complex composed of activated factors FVIII/FIX, the latter having two factors missing or deficient in persons with HA/HB [3]. Without the amplification phase, the attenuation of TFPI inhibition can restore thrombin generation [44]. The idea of deploying TFPI inhibition in the treatment of HA and HB dates back to 1991 [45].

## 4. Concizumab: Mechanism of Action

**Figure 1:**
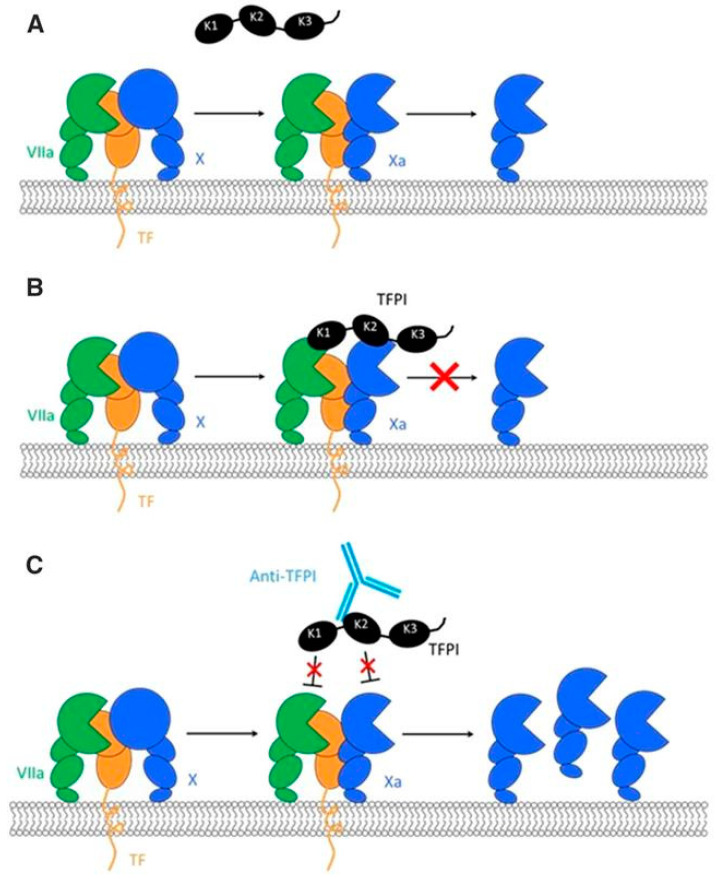
Mechanism of action of tissue factor pathway inhibitors (TFPIs), such as concizumab (reproduced from [45]). (**A**) Tissue factor-based initiation of coagulation and generation of activated factor X (Xa) by the extrinsic tenase complex. (**B**) Inhibition of Xa and activated factor VIIa (VIIa) by TFPI. (**C**) Binding of the different Kunitz (K) domains by the various anti-TFPI antibodies. X, factor X.

Concizumab is a humanized monoclonal immunoglobulin G4 antibody against TFPIs that binds to the Kunitz-2 domain of TFPIs and prevents TFPIs from directly binding to FXa and from indirectly binding to the tissue factor/FVIIa complex (Figure 1); the result is increased thrombin generation through the extrinsic pathway [46]. Concizumab binds to both soluble and membrane-bound TFPIs with high affinity, contributing to non-linear pharmacokinetics (i.e., target-mediated drug disposition [TMDD]) [47]. TMDD, which can be considered a consequence of pharmacodynamics affecting pharmacokinetics [48], describes a situation in which a large proportion of an administered drug dose binds with high affinity to its target, contributing to significant drug elimination until the saturation of target binding. At low drug concentrations, the administration of increasing doses is associated with an apparent decrease in the steady-state volume of distribution until the target is saturated [45]. The impact of TMDD on the pharmacokinetics of concizumab was studied in Cynomolgus monkeys and showed that the terminal half-life depended on the plasma concentration [49]. Phase 1 studies confirmed that the concizumab half-life ranged from 31.1 to 74.2 h and depended on the plasma concentration and route of administration [47]. At a steady state, following multiple subcutaneous injections, the estimated half-life is approximately 38 h [50]. Such a short half-life is an advantage when the discontinuation of concizumab administration is needed, as a quick washout is attained.

The hemostatic effect of concizumab was first confirmed in vitro and in a rabbit model of hemophilia [46] and, later, in the first human trial [47]. In all trials, the concizumab plasma concentration correlated with increased thrombin generation and elevated levels of fibrin D-dimer and prothrombin fragments 1 + 2. Given that fibrin D-dimer and prothrombin fragments 1 + 2 are biomarkers of coagulation activation [51,52], their elevated levels proved the hemostatic effect of concizumab [47,53,54].

Mechanistically, concizumab does not affect the downstream regulation of coagulation and can be combined with treatments for breakthrough bleeds [55,56]. Such bleeds in persons with HA or HB on concizumab prophylaxis can be treated with bypassing agents (e.g., aPCC, recombinant FVIIa [rFVIIa]), or with recombinant FVIII (rFVIII) or FIX (rFIX), depending on each patient’s inhibitor status. In the presence of concizumab, rFVIIa, aPCC, rFVIII, and rFIX enhance the plasma thrombin generation potential, and available in vitro data support their use to treat mild and moderate breakthrough bleeds in patients on concizumab. Dosing during on-demand treatment needs to be adjusted to ensure patient safety (i.e., the lowest approved dose, as per label, should be administered) [56]. Concizumab has no antidote, but it has the advantage of a quick washout [57].

The product characteristics of concizumab are summarized in Table 1.

## 5. Efficacy and Safety of Concizumab in Persons with HA or HB with Inhibitors

Concizumab efficacy in persons with HA or HB with inhibitors was evaluated in the explorer4 and 7 trials [53,54,59]. The explorer4 trial was a successful, phase 2, proof-of-concept trial in persons with HA or HB with inhibitors [45,59]. The primary objective of explorer4 was to assess the efficacy of once-daily concizumab in preventing bleeding episodes in persons with HA or HB with inhibitors; efficacy was evaluated as the number of bleeding episodes during at least 24 weeks from treatment initiation. Starting with a maintenance dose of 0.15 mg/kg, the dose was escalated to 0.20 mg/kg and then to 0.25 mg/kg in patients who had ≥3 treatment-requiring spontaneous bleeding episodes within the 12 weeks before concizumab treatment, during both the main and extension study parts [59]. The estimated ABR during the main and extension study parts at the last dose level was 4.8 (95% confidence interval [CI]: 3.2–7.2; median ABR 3.6), and for spontaneous bleeds was 1.8 (95% CI: 1.2–2.6). Importantly, switching from no prophylaxis in the main study part to prophylaxis with concizumab in the extension part decreased the estimated ABR from 18.6 (95% CI: 12.9–26.9) to 4.9 (95% CI: 2.2–10.6) [54].

explorer7 was designed as a phase 3 safety and efficacy trial in persons with HA or HB with inhibitors [53]. The primary endpoint compared the number of treated spontaneous and traumatic bleeding episodes in group 1 (no prophylaxis for at least 24 weeks) with the number in group 2 (prophylaxis with concizumab for at least 32 weeks) [53]. The estimated mean ABR ratio for treated spontaneous and traumatic bleeding episodes between group 1 and group 2 was 0.14 (95% CI: 0.07–0.29), confirming the superiority of concizumab prophylaxis over no prophylaxis. The median ABR was 9.8 (interquartile range [IQR] 6.5–20.2) episodes in group 1 (i.e., estimated mean ABR 11.8; 95% CI: 7.0–19.9) and 0.0 (IQR 0.0–3.3) episodes in group 2 (i.e., estimated mean ABR 1.7; 95% CI: 1.0–2.9). The overall median ABR for patients receiving concizumab (groups 2, 3, and 4) was 0.0 (IQR 0.0–3.3) episodes [53].

Joint health outcomes are an important aspect of any treatment modality used in persons with hemophilia. The explorer4 and 7 trials demonstrated good protection against joint bleeds for persons with HA or HB with inhibitors [53,54,59]. In explorer4, a joint ABR of 2.7 (95% CI: 1.6–4.6) was observed. Moreover, the estimated mean joint ABR decreased from 13.8 (95% CI: 9.6–19.9) to 2.9 (95% CI: 1.1–7.7) in patients switched from no prophylaxis to prophylaxis with concizumab [54]. explorer7 further confirmed that concizumab prophylaxis helped joint health, as the median ABR for joint bleeding episodes was higher in group 1 than group 2 (6.5 [IQR 3.2–13.1] vs. 0.0 [IQR 0.0–2.6]); the median ABR for target joint bleeding episodes was 0.0 (IQR 0.0–2.2) in group 1 versus 0.0 (IQR 0.0–0.0) in group 2 [53]. Also, concizumab prophylaxis resolved 91.8% of target joints (i.e., joints with recurrent bleeding) in persons with HA or HB with inhibitors, usually within 12 months. The median ABR for treated spontaneous and traumatic target joint bleeding episodes at 56 weeks in persons with HA or HB with inhibitors was 0.0 [60].

Equally important is the performance of bleeding prophylaxis in persons with hemophilia undergoing scheduled surgery. The results from phase 3 trials showed that minor surgeries could be performed in patients receiving concizumab prophylaxis. A total of 11% of patients (30/278) in those trials had minor surgical, mostly dental, procedures. Surgery-related bleeding occurred in 24 patients: most bleeding episodes were mild or moderate, and 17 episodes were treated in 15 patients [61].

Safety was the primary endpoint in the explorer1, 2, and 3 trials, during which adverse event (AE) analysis confirmed the safety of concizumab: all reported AEs were mild, and there were no serious AEs [47,62,63]. In the proof-of-concept trials, during both the main and extension parts, most AEs were mild, with no deaths, no events leading to withdrawal, and no thromboembolic events [54,59]. There was, however, a serious safety issue that led to a pause of the explorer7 study. Three patients on concizumab, including one patient from the explorer7 trial, and all with thrombotic risk factors at the baseline, experienced nonfatal thromboembolic events, which included a renal infarct in one patient. All three patients were receiving concomitant hemostatic medication before or on the day of their thromboembolic event and two patients were at the high end of the concizumab exposure range in phase 3 trials [64]. Following a careful analysis of the available data from the phase 2 and 3 trials, a risk-mitigation strategy was implemented and consisted of guidelines on the concomitant use of hemostatic agents in the treatment of bleeding episodes while on concizumab prophylaxis, and updated concizumab dosing (i.e., the daily maintenance dose was lowered from 0.25 to 0.20 mg/kg, with the dose adjustment based on concizumab plasma concentrations within the initial 5–8 weeks of treatment). The explorer7 study was later resumed with an amended protocol, and no thromboembolic events were observed after the implementation of the risk-mitigation strategy [53]. Post-authorization pharmacovigilance is also very important in hemophilia research [65]. Such efforts should be made for all available treatments and for products in late-stage clinical development, as in the future, these efforts will help associate patient profiles with the most appropriate treatments.

A summary of the results from the explorer clinical development program, with a focus on persons with HA or HB with inhibitors, is shown in Table 2.

## 6. Adherence to Treatment

Despite the undeniable benefits of prophylaxis with replacement factors, adherence to this treatment modality has always been a significant problem due to barriers discussed elsewhere [6,66]. It is expected that less burdensome non-replacement together with the patient-oriented choice of the optimal treatment based on shared decision making between patients and physicians will improve adherence [6,67].

Concizumab prophylaxis is delivered as a daily subcutaneous injection administered through a pre-filled multidose pen, much like the pens used by persons with diabetes on insulin [7]. In a Canadian study of time trade-off utilities, prophylaxis delivered subcutaneously was associated with higher utility values than intravenous prophylaxis or on-demand treatment [68]. In chronic diseases, once-daily treatment was linked to higher adherence rates than more frequent medicine administration [69]. In the clinical trials, the daily administration of concizumab was not associated with poor adherence [53,54,59]. Concizumab is self-injected or injected by a caregiver. Although unlikely erroneous intramuscular administration is a possibility, especially in thin individuals, with adequate training, patients or their carers easily learn how to perform the injections [50]. Moreover, providing patients with device training and the concurrent teaching/development of routines or rituals may be useful to further improve adherence [70]. In the explorer7 trial, over 90% of patients who responded to the Hemophilia-Patient Preference Questionnaire preferred concizumab over their previous treatment [53]. The three main reasons for the preference for concizumab were “fewer bleeds” (75%), “require less time” (43%), and “less painful to inject” (33%) [71]. A similar rate of preference (88%) was reported by a subsequent study. In this study on the patient and caregiver preference between the pre-filled concizumab pen or current systems to deliver prophylaxis, 98% of participants considered the pre-filled pen ‘easy’ or ‘very easy’ to use. The pen gave participants high confidence in a full dose being delivered correctly and reduced anxiety as the needle is only 32G and 4 mm long [72,73]. In addition, the precise weight-based dosing of concizumab, with its specialized pen, will avoid the product wastage encountered with emicizumab [74].

## 7. Outcome Measures

The development of novel therapies has markedly reduced the burden of hemophilia and its treatment. However, novel outcome measures are needed and potential outcome measures in hemophilia have been reviewed [75]. To evaluate the outcomes of treatment in persons with a near-normal life expectancy, and the lower disease burden (such as bleeding rates approaching zero) and lower treatment burden (resulting in an improved quality of life), objective clinical methodologies should be combined with patient-reported outcomes [75].

The bleeding phenotype, joint health status, physical activity expectations, drug half-life and properties, and capacity to adhere to treatment should guide the optimization of prophylaxis in hemophilia to secure the best outcomes [9,67]. However, joint health assessments with adequate sensitivity and specificity continue to be an unmet need [76]. Incidentally, adults with hemophilia with inhibitors have more severe and rapidly progressing hemophilic arthropathy than those without inhibitors [77]. This underlines the importance of effective joint health assessments in this patient category. A point-of-care ultrasound for joints could help in the early detection of arthropathy and monitor its progression, together with assessing the treatment efficacy [76].

Moreover, health outcomes that are important for persons with HA or HB should not be neglected [75]. Knowing what matters to patients is essential for delivering patient-centered care. Patients’ perceptions of joint damage, pain, and the impact of hemophilia and its treatment on daily living can best be gauged using patient-reported outcome measures. One new tool for assessing the disease and treatment burden from patients’ perspectives is the Hem-TEM tool [78]. Obtaining a good health-related quality of life in hemophilia should be considered a new therapeutic goal [79]. Patient-reported outcomes in the explorer7 study showed improvements favoring concizumab in the health-related quality-of-life domains of ‘feeling’, ‘treatment’, ‘view of yourself’, and ‘sport and leisure’. Improvements were also perceived regarding the treatment burden and patients’ treatment preferences in persons with HA or HB with inhibitors during concizumab prophylaxis [80].

## 8. Discussion

There has been enormous progress over the years in the management of HA and HB. Persons with HA or HB who were once dying of bleeding, or subsequently of AIDS after the transfusion of blood-derived factors, now have a near-normal life expectancy [81]. Traditionally, there were limited options for persons with HB with inhibitors [23]. However, with the advent of rebalancing agents, such as concizumab, this gap is now being filled. The development of TFPIs including concizumab also provides exciting opportunities for persons with HA with inhibitors, especially those unable to attain adequate hemostasis and experience bleeding episodes during treatment with currently approved modalities. Concizumab was the first of its class to be approved, but other TFPIs are being developed and approved.

Concizumab is effective (improved ABRs and patient-reported outcomes) and delivered in a way that patients prefer, which will probably enhance adherence to treatment [53,72]. Furthermore, the explorer clinical development program showed that accurate data analysis and appropriate risk-mitigation and safety measures can ward off the potential for thrombosis seen in earlier trials [53,64]. This is not to say that dose-lowering alone is sufficient to eliminate the risk of thromboembolic events and patient safety will have to be ensured also with the appropriate monitoring.

There is currently no established consistent approach to monitor the treatment with concizumab. The measurement of TFPIs, given their very short half-life in circulation, especially of the plasma fraction, is difficult [82]. Moreover, the measurement of plasma TFPIs may not be a reliable indicator of the anticoagulant activity of TFPIs present in an individual patient [83]. For concizumab and other rebalancing agents, global assays are an option but, to date, none have been routinely used in monitoring. The thrombin generation assay (TGA) is a versatile global assay for the evaluation of the hemostatic balance that has been employed extensively to monitor the hemostasis in patients with hemophilia [84]. The TGA remains difficult to standardize; however, efforts are being made to improve this aspect [85]. A recent paper confirmed that the plasma presence of concizumab only marginally alters the results of global assays, highlighting that the TGA is a viable option [86]. Clearly, the appropriate monitoring of treatment with concizumab is needed to avoid thromboembolic events.

Additional real-world evidence is now being acquired for concizumab, which is already available in five countries, and it will be extremely important to consider the inclusion of concizumab, together with other innovative therapies (including other TFPIs, i.e., the recently approved marstacimab and, once approved, befovacimab or antithrombin-lowering therapy, fitusiran), in national and international guidelines on hemophilia management as soon as possible.

Where will the hemophilia research be in 5 or 10 years’ time? One could ask whether, by concentrating all research efforts in hemophilia management on gene therapy, it is hoped to provide an ultimate cure. At present, gene therapy is not available in persons with inhibitors, although some studies have shown waning inhibitor titers following liver-directed gene therapy in animal models [87]. There are also ethical implications of gene therapy: e.g., the available products may not yet provide a permanent cure, or the inaccessibility of gene therapy to most persons with hemophilia because of the cost [88]. Thus, there is a need (continuing into the foreseeable future) for all available treatments, including concizumab.

The limitation of this review is the lack of real-life reports on the use of concizumab. In hemophilia, which is a rare disease, conducting prospective clinical trials and observational studies with statistically meaningful endpoints is difficult, which underlines the importance of the real-life setting [89]. However, the real-life reports are still not available.

Clearly, the management of hemophilia will have to become personalized. As our understanding of genetic and phenotypic variations in hemophilia deepens, treatments will increasingly be tailored to individual patient profiles, needs, preferences, and expectations [90]. The ultimate goals in the field are to improve clinical and patient-reported outcomes for persons living with hemophilia, and to provide normal and fulfilling lives for persons with this condition worldwide.

## 9. Conclusions

Due to its unique mechanism of action, concizumab is the first prophylactic treatment approved for persons with HB with inhibitors. It represents a promising therapeutic option for persons with HA with inhibitors who continue to experience bleeds during prophylaxis with emicizumab. Since 2023, concizumab has been approved in Canada, Australia, Japan, and Switzerland, and in France through early-access authorization [50,91,92,93,94]. The development of a non-replacement treatment modality (e.g., concizumab), with a convenient mode of subcutaneous administration using a multi-dose pen, offers a chance at a normal life to persons with HA or HB with inhibitors, who have traditionally had a long history of a compromised health-related quality of life; moreover, the short half-life and quick washout of concizumab make this therapy a highly adaptable treatment option.

## Figures and Tables

**Table 1:** Summary of product characteristics of concizumab.

Characteristic	Description
Mechanism of action [45]	Concizumab binds to the Kunitz-2 domain of the TFPI protein and prevents TFPI from binding to FXa and to the TF/FVIIa complex; the inhibition of TFPI increases thrombin generation
Administration [50]	Subcutaneous using a prefilled multidose pen
Half-life [50]	38 h ^a^
Frequency of administration [50]	Once daily
Dose calculation [50]	Patient bodyweight (kg) × dose (1.00, 0.15, 0.20 or 0.25 mg/kg) = total amount (mg) of concizumab to be administered in a single daily injection
Antidote [57]	None, but quick washout
Laboratory monitoring ^b^ [58]	Monitoring drug concentration: Measurement of TFPI levels using ELISA Measurement of residual TFPI activity using specific activity assays, e.g., diluted PT-based assay or TF-dependent chromogenic assays Monitoring drug efficacy: thrombin generation, thromboelastography, clot waveform analysis before and after treatment commencement
Breakthrough bleed treatment	No concizumab dose adjustment needed Bypassing agents (rFVIIa, aPCC, plasma-derived FVIIa/FX), factor concentrates
Laboratory monitoring during concomitant treatment with concizumab and bypassing agents	Thrombin generation
Treatment management during surgery	Minor surgery: no concizumab dose adjustment needed Major surgery: concizumab should be paused 4 days prior to surgery and resumed at the normal daily maintenance dose (either 0.15, 0.20, or 0.25 mg/kg) 10–14 days after surgery, considering each patient’s overall clinical picture ^c^
Immunogenicity [54]	In the explorer4 and 5 trials [54], 25% of patients developed mostly low-titer and transient neutralizing anti-concizumab antibodies
AEs (frequency in the explorer7 trial) [53]	Common AEs (occurring in ≥5% of patients): injection-site reactions (22.8%), arthralgia (11.4%), upper respiratory tract infections (7.0%), headache (5.3%), pyrexia (5.3%) Less common AEs: hypersensitivity (2.6%), thromboembolic events (0.9%), pruritus (0.9%)

^a^ Half-life depends on plasma concentration and route of administration. ^b^ In the explorer7 trial [53], concizumab concentration, free TFPI concentration, and thrombin peak were measured. ^c^ Patients with planned surgery were excluded from the concizumab studies. AE, adverse event; aPCC, activated prothrombin complex concentrate; ELISA, enzyme-linked immunosorbent assay; FVIIIa, activated factor VIII; FX, factor X; FXa, activated factor X; PT, prothrombin time; rFVIIIa, recombinant activated factor VIII; TF, tissue factor; TFPI, tissue factor pathway inhibitor.

**Table 2:** Summary of the explorer trials.

Trial ID	Study Type	Intervention	Number of Participants	Findings
explorer1 (NCT01228669) Phase 1	A multicenter, randomized, double-blind, placebo-controlled, single-dose, dose-escalation trial investigating safety, PK and PD of NNC 0172-0000-2021 administered intravenously, and SC to healthy male subjects and persons with HA or HB	Concizumab or placebo	52 (28 healthy volunteers, 24 persons with HA or HB)	Primary endpoint: safety 76 AEs (75% mild)
explorer2 (NCT01631942) Phase 1	A multicenter, open-label, multiple-dosing trial investigating safety, PK and PD of NNC 0172-2021, administered SC to healthy male subjects and persons with HA or HB	Low, medium, or high dose of concizumab	22 (4 healthy volunteers, 18 persons with HA or HB)	Primary endpoint: safety No severe or unexpected AEs Increased thrombin generation with concizumab in thrombin generation assay ex vivo and in vivo
explorer3 (NCT02490787) Phase 1b	A multicenter, randomized, placebo-controlled, double-blind, multiple-dose trial investigating safety, PK, and PD of concizumab, administered SC to persons with HA	Placebo, or five escalating doses of concizumab	24	56 AEs in 19 persons (54 mild and 2 moderate); 91 bleeds (almost all mild)
explorer4 (NCT03196284) Phase 2	A multicenter, randomized, open-label, controlled trial evaluating the efficacy and safety of prophylactic administration of concizumab in persons with HA or HB with inhibitors	Concizumab (main and extension phases), with eptacog alfa administered on-demand during bleeding episodes	26	Estimated ABR 4.5 (95% CI: 3.2–6.4) in the concizumab arm vs. 20.4 (95% CI: 14.4–29.1) in the rFVIIa on-demand arm Low AE rates, no severe AEs reported, no AE-related withdrawals, no thromboembolic events, and no deaths
explorer6 (NCT03741881) Phase 3	A prospective, multinational, non-interventional study in persons with HA or HB with or without inhibitors treated according to routine clinical practice	No treatment given	231 *	No published results
explorer7 (NCT04083781) Phase 3	Efficacy and safety of concizumab prophylaxis in persons with HA or HB with inhibitors	No prophylaxis for ≥24 weeks (group 1), prophylaxis with concizumab for ≥32 weeks (group 2), or nonrandomly assigned to prophylaxis with concizumab for ≥24 weeks (groups 3 and 4)	133 (19 in group 1; 33 in group 2; 21 in group; and 60 in group 4)	Median ABR was 9.8 (IQR 6.5–20.2) in group 1 vs. 0.0 (IQR 0.0–3.3) in group 2 Overall median ABR in the concizumab groups was 0.0 No thromboembolic events after resuming the therapy

* Enrolment as of November 2015. ABR, annualized bleeding rate; AE, adverse event; CI, confidence interval; HA, hemophilia A; HB, hemophilia B; IQR, interquartile range; PD, pharmacodynamics; PK, pharmacokinetics; rFVIIa, recombinant activated factor VII; SC, subcutaneously.

## Abbreviations

The following abbreviations are used in this manuscript:

ABR	Annualized bleeding rate
AE	Adverse event
aPCC	Activated prothrombin complex concentrate
CI	Confidence interval
ELISA	Enzyme-linked immunosorbent assay
FIX	Factor IX
FVIIa	Activated factor VII
FVIII	Factor VIII
FX	Factor X
HA	Hemophilia A
HB	Hemophilia B
IQR	Interquartile range
rFIX	Recombinant factor IX
rFVIII	Recombinant factor VIII
TF	Tissue factor
TFPI	Tissue factor pathway inhibitor
TMDD	Target-mediated drug disposition
